# The Fineness of Gold Solders

**Published:** 1906-09-15

**Authors:** S. H. Guilford


### THE FINENESS OF GOLD SOLDERS.

S. H. GUILFORD, A.M., D.D.S., PH.D.

In these days when those who have the best interests of the profession at heart are endeavoring to establish a scientific exactness in its various departments, it is discouraging to notice an opposite tendency on the part of some of those who furnish the supplies for our handicraft.

The writer, along with others, has observed this in referto the misleading way in which nearly all manufacturers at present stamp their gold solders.

Gold plate is priced and sold at its actual karat value, and gold solders used to be but are not now.

Formerly gold solders were stamped with the karat mark. Eighteen-karat solder meant solder containing eighteen parts of pure gold to six of alloy, and eighteen-karat gold plate meant the same, the difference being in the character or the alloying metals, the solder containing some lower fusing metal that lowered the fusing point of the mass. Still both being of the same karat, one was as fine as the other.

Now while the eighteen-karat solder could be used on an eighteen-karat plate because of its lower fusing ingredient, it was attended with some risk; so the student in private laboratory or in college was taught to use a solder two karats lower than the plate. This rule has been laid down and followed for half a century or more and it was satisfactory because it was safe and exact. Everybody understood it.

Within the past few years, however, a change has been creeping in, until now the karat mark is no longer used to designate the fineness of gold solders. Instead of being stamped "18 k." the envelopes containing the dwt. pieces are printed "for 20 k. plate." The change may seem a small one, but it is great nevertheless for two reasons.

1. The purchaser does not know what he is buying.

A solder marked "for 20 k. plate" may be 18 karats fine or it may be 16 or 14 or 12. Nobody knows but the manufacturer, and he doesn't tell. Even if assayed and shown to be but 16 or 17 karats fine it might still truthfully be "for 20 k. plate."

It is indefinite, and to that extent misleading.

2. It is a temptation to dishonesty, for while some manufacturers would doubtless make a solder 18 karats fine to be used with 20 k. plate, however it might be marked, certain other ones would probably lower the karat of their solders and hide behind the screen "for —k. plate."

A few years ago, after one or two manufacturers had changed their method of marking their solders the writer's attention was drawn to a significant statement in a pamphlet issued by a manufacturer of gold plate and solders. It read as follows:—"We sell our solders at their value, on their merits and not by means of misleading karat stamps."

This looked as though somebody had been marking his solders with a higher karat stamp than they deserved. The suspicion seemed to be confirmed by the following from the same pamphlet:

| OUR SOLDERS. | | A. B. C. CO'S SOLDERS. | | |
|---|---|---|---|---|
| Stamped. | Fineness. | Stamped. | Actual Karat. | Fineness. |
| 22 | 833–1000 | 22 kt. | 18 kt | 754–1000 |
| 20 | 750–1000 | 20 kt. | Less than 18 kt. | 737–1000 |
| 18 | 680–1000 | 18 kt. | Less than 16 kt. | 657–1000 |
| 16 | 572–1000 | 16 kt. | Less than 14 kt. | 550–1000 |
| 14 | 482–1000 | 14 kt. | Less than 12 kt. | 439–1000 |

"In addition to these comparative tables of fineness we have on exhibit in our office United States assay certificates showing fineness of all other well-known makes of dental gold solders."

Only a month or more ago the writer noticed an advertisement appearing in several dental journals headed "18 karat dark, gold solder.

Feeling satisfied that this was a definite statement as to fineness, an order was sent for several dwt's, enclosing check, accompanied by some words of commendation for marketing a solder with its real karat indicated. The solder came, but with it the following statement:

"We note your remarks in regard to this being real 18 k., and wish to state that it is not marked 18 k., but 'for' 18 k. work. However, we could make you a strictly 18 k. solder. In fact, for years we put our solder out up to the standard, until we found other manufacturers selling theirs at a lower price, it being a lower karat." The solder was returned and the check came back.

Here seemed to be an explanation of the cause of the *downfall.* Competition induced someone to retrograde and the others followed.

Wishing to know how manufacturers in general felt about the change from the original karat stamp, and desiring to afford them an opportunity to declare the real fineness of their solders, a letter was addressed to seven of the most prominent ones embodying the above queries. It contained a request for them to furnish for publication (if they would) the fineness, either in karats or thousandths, of the solders they sold to be used with 22, 20, 18 and 16 karat plate.

All replied, but only two gave the actual fineness of their solders. One said: "Referring to your inquiry regarding, solder, we beg to say that ours is not 18 karat fine, but is the same as others, and made to be used on 18 k. plate."

Another: "I think that formerly the karat mark on solders represented more accurately the karat, to correspond with plate, than it does at present. I attribute this change to the cheapening of the retail price. My solders are two karats less than the plate on which they are to be used."

Another: "As to our gold solders would say that they are not and never were marked with their karat, and we never claimed that they were. The stamp 22, 20, 18, 16 or 14

indicates the grade of gold same are intended to be used for. It is *our* firm, who *first* called the dentist's attention to the misrepresentation practiced on that score, showing at the same time that our solders are higher in grade and quality than any other makes."

(This is the firm that published the comparative tables shown on a preceding page.)

Still another says: "In your reply to your very courteous letter would say that gold solders have *always* been and are now two karats below the plates they are intended for, otherwise in the hands of inexperienced or careless users it migh 'burn' or destroy the texture of the plate before the solder would flow.

Here is a reply less polite than the others and embodying a misconception, for we neither asked for nor desired any formulæ. We only requested a statement as to actual fineness.

"It has been the custom for a great many years to designate the kind of plate the solder is to be used on. This is done mainly in our case to prevent the dentists from making mistakes. We do not care to supply you with the formula for our gold solders. If you wish to find out the percentage of gold which they contain, this can be accomplished very easily by having them analized for gold."

It is hard for us to conceive how dentists could make a *mistake* in using a *real* 18 k. solder on a 20 k. plate. It seems much more likely that a mistake would be made in using an indefinite solder on any plate, for while the soldering in the first instance might be entirely successful, in any subsequent soldering by someone else (as for repairs) he would be at a loss to know what grade of solder to use.

The last reply said: We hereby give you the amount of pure gold contained in our 16, 18 and 20 karat solders."

| | |
|---|---|
| 16 karat solder | 55.76 percent. |
| 18 karat solder | 65.98 percent. |
| 20 karat solder | 73.80 percent. |

As in this case each of the solders mentioned is approximately 2 karats less than 16, 18 and 20 respectively, it would seem as though our informant were giving us the fineness of solder "for 16 (or 18 or 20) plate" instead of the actual fineness of the solders themselves.

However, in the course of some explanatory remarks, he says: "The 18-karat solder appearing on this list is what has been termed our regular" 18-karat solder for many years."

As some years ago all manufacturers stamped their solders with the supposed real karat mark, we cannot make out whether the above "many years" covered this period or not.

From what has been written we may fairly conclude that most of those who replied to our queries are placing upon the market solders that are approximately two karats lower in fineness than the plates upon which they are to be used, but whether all others are doing the same we have no means of knowing.

It will be noticed that two reasons are given for the change in the manner in stamping the solders. One is, to prevent the inexperienced or careless from overheating the plates in soldering; and the other, that some one made the change for purposes of deception and the rest had to follow suit in order to avoid losing their trade.

As previously explained, marking solders with their real karat mark would not only not mislead any one but it would prove a distinct advantage in enabling one to know what he is using.

In regard to the change being made "because others did it," it would seem to be a poor policy to pursue, for those who put the real karat mark upon their solders would show to the world their sincerity in producing a material fully equal to the claim made for it. Any possible deception would be avoided.

Manufacturers are justly proud of the stamp or trademark placed upon their other goods, claiming that it represents the real article and the best that they can turn out. We cannot see the wisdom or justice in not following this rule in regard to gold solders, and we certainly believe that if a return were made to the former plan of stamping them with the actual karat mark, both the manufacturers and the profession would be the gainers.—*Stomatologist.*

